# *BDNF* Variants May Modulate Long-Term Visual Memory Performance in a Healthy Cohort

**DOI:** 10.3390/ijms18030655

**Published:** 2017-03-17

**Authors:** Nesli Avgan, Heidi G. Sutherland, Lauren K. Spriggens, Chieh Yu, Omar Ibrahim, Claire Bellis, Larisa M. Haupt, David H. K. Shum, Lyn R. Griffiths

**Affiliations:** 1 Genomics Research Centre, Chronic Disease and Ageing, Institute of Health and Biomedical Innovation, School of Biomedical Sciences, Queensland University of Technology, Brisbane 4059, Australia; nesli.avgan@qut.edu.au (N.A.); heidi.sutherland@qut.edu.au (H.G.S.); c22.yu@hdr.qut.edu.au (C.Y.); omarezzedin@gmail.com (O.I.); bellisc@gis.a-star.edu.sg (C.B.); larisa.haupt@qut.edu.au (L.M.H.); 2 Menzies Health Institute Queensland and School of Applied Psychology, Griffith University, Gold Coast 4222, Australia; l.spriggens@griffith.edu.au (L.K.S.); d.shum@griffith.edu.au (D.H.K.S.); 3 Human Genetics, Genome Institute of Singapore, Agency for Science, Technology and Research of Singapore, Singapore 138672, Singapore

**Keywords:** BDNF, long-term visual memory, human memory, genetics of memory

## Abstract

Brain-derived neurotrophic factor (BDNF) is involved in numerous cognitive functions including learning and memory. BDNF plays an important role in synaptic plasticity in humans and rats with BDNF shown to be essential for the formation of long-term memories. We previously identified a significant association between the *BDNF* Val66Met polymorphism (rs6265) and long-term visual memory (*p*-value = 0.003) in a small cohort (*n* = 181) comprised of healthy individuals who had been phenotyped for various aspects of memory function. In this study, we have extended the cohort to 597 individuals and examined multiple genetic variants across both the *BDNF* and *BDNF-AS* genes for association with visual memory performance as assessed by the Wechsler Memory Scale—Fourth Edition subtests Visual Reproduction I and II (VR I and II). VR I assesses immediate visual memory, whereas VR II assesses long-term visual memory. Genetic association analyses were performed for 34 single nucleotide polymorphisms genotyped on Illumina OmniExpress BeadChip arrays with the immediate and long-term visual memory phenotypes. While none of the *BDNF* and *BDNF-AS* variants were shown to be significant for immediate visual memory, we found 10 variants (including the Val66Met polymorphism (*p*-value = 0.006)) that were nominally associated, and three variants (two variants in *BDNF* and one variant in the *BDNF-AS* locus) that were significantly associated with long-term visual memory. Our data therefore suggests a potential role for *BDNF*, and its anti-sense transcript *BDNF-AS*, in long-term visual memory performance.

## 1. Introduction

Human memory is a complex neurocognitive and polygenic trait with different memory systems responsible for its encoding-retaining-retrieving abilities [1,2]. The traditional memory model consists of three parts—sensory memory (SM), short-term memory (STM, also known as working memory), and long-term memory (LTM)—and was named as “the modal model” by Richard Atkinson and Richard Schifrin in 1968 [3]. The shortest-term element of memory is SM, which holds information after a stimulus is received through the five senses: sight, hearing, smell, taste, and touch. Sight, the visual element of SM, is named as iconic memory [4]. When the information held by SM becomes more durable it is transferred to STM. However, STM has a limited capacity and a limited time frame [5,6]. Repetitive information might later be transferred to LTM, which has a larger capacity and covers larger time frames to store lasting information [7].

Our knowledge of the pathways that regulate memory, along with the genes and molecules playing a role in the formation-storage-retrieval processes, is still limited. However, genes and polymorphic markers identified in recent studies have provided candidates to be further investigated in different population cohorts, and one of these is brain-derived neurotrophic factor (BDNF) [8,9,10,11]. *BDNF* encodes the BDNF protein which is expressed in the brain and regulates synaptic plasticity in humans [12]. Synaptic plasticity involves strengthening or weakening of the synapses in response to their activity, and memory formation takes place with this process [13]. Thus, synaptic plasticity has a central role in nearly all models of learning and memory [14]. BDNF stimulates long-term potentiation in the hippocampus, which is a type of synaptic plasticity that mediates LTM formation [15,16]. In rat studies, BDNF was shown to regulate short-term synaptic function and activity-dependent synaptic plasticity, which is required for LTM formation.

The human *BDNF* gene located on chromosome 11 produces multiple transcripts and is expressed in a diverse range of tissues, with high levels in the central nervous system [17]. *BDNF* produces a precursor protein (proBDNF) that is proteolytically cleaved to form mature BDNF [18]. ProBDNF preferentially binds to the receptor p75NTR which can trigger apoptosis, axonal retraction, and pruning of dendritic spines, while mature BDNF binds to the TrkB receptor tyrosine kinase to mediate the cell cycle, neurite outgrowth, and synaptic plasticity [19]. The gene is also regulated by a non-coding BDNF antisense RNA gene (*BDNF-AS*) that is positioned downstream of *BDNF*. *BDNF-AS* transcription can repress *BDNF*; it has been reported that inhibition of *BDNF*-*AS* upregulates *BDNF* mRNA, which subsequently increases protein levels and stimulates neuronal outgrowth and differentiation [17,20,21]. Therefore, both *BDNF* and *BDNF-AS* may influence cognition and learning and are good candidate loci for investigating the impact of genetic polymorphisms on aspects of memory performance in humans.

G196A (known as rs6265) is a well-studied non-synonymous G to A single nucleotide polymorphism (SNP) in the *BDNF* gene. It is in the pro-protein region of BDNF and results in substitution of valine for methionine residue at position 66 (Val66Met). This affects the intracellular packaging of proBDNF and activity-dependent secretion of the mature form of BDNF, and has been found to be associated with poorer verbal episodic memory scores and many other cognitive functions [22]. rs6265 has also been reported to be associated with regional gray matter volume in the literature [23,24], however, meta-analyses of the *BDNF* Val66Met polymorphism for its association with hippocampal volume by Harrisberger et al. failed to find associations in either healthy cohorts or individuals diagnosed with neuropsychiatric disorders [25,26]. Another meta-analysis performed in 2012 by Mandelman and Grigorenko explored the *BDNF* Val66Met polymorphism and cognition [27]. Although *BDNF* Val66Met has been associated with several cognitive functions, this meta-analysis also failed to support significant associations between the SNP and cognition, memory, executive function, and visual and cognitive fluency phenotypes [27]. To comprehensively study the role of *BDNF* polymorphisms in cognitive and memory functions it may be important to investigate variations in a larger region of the *BDNF* locus, rather than focusing on just the Val66Met polymorphism, since transcription and regulation at the locus is complex.

We previously investigated the role of the *BDNF* Val66Met polymorphism in a small cohort (*n* = 181) that had been phenotyped for a range of human memory sub-types. Our results demonstrated significant effects of *BDNF* on long-term visual memory (*p*-value = 0.003), and we found that the Val/Val genotype was linked with poorer visual memory [28]. In the present work, we extended our investigation of variants involved in visual memory performance to 38 SNPs in both the *BDNF* and *BDNF-AS* genes in a larger cohort of healthy individuals (*n* = 597). We found that a number of SNPs that are nominally associated and three SNPs in both genes that are significantly associated with long-term visual memory.

## 2. Results

### 2.1. Demographics and Phenotype Analysis

Statistical analysis was performed with a cohort of 597 healthy individuals who had undertaken a battery of memory tests. Demographics of the memory cohort are presented in Table 1. Two thirds of the cohort was female (71%). The age of participants ranged from 16 to 65 years (M = 20, SD = 8.57), and three quarters of the cohort identified as Caucasian. Due to the large number of individuals with other ethnicities (*n =* 151), population structure was studied and added to the analysis as covariates along with age and gender.

Correlation analysis showed that intelligence quotient (IQ) was not correlated with any of the memory tests, and it was added into the analysis as another covariate. In this study, visual memory performance was measured using the Wechsler Memory Scale—Fourth Edition (WMS IV) subtests Visual Reproduction I for immediate visual memory (VR I) and Visual Reproduction II (VR II) for long-term visual memory. VRI and II are widely used psychological tests for clinical assessments and in research. To assess VR I, each examinee is asked to draw geometrical patterns they have just been shown, immediately and in any order. Following a delay period, participants are asked to draw the previously shown patterns from their memory to obtain the VR II score. When we examined the scores of the VR I and VR II subtests we found that they were moderately correlated (0.62) with each other. This may reflect the nature of the tests as VR II repeats the same visual components of the VR I test, but with a delay component.

### 2.2. Genotype Association

Visual memory phenotypes obtained using the VR I and VR II subtests of WMS IV were analyzed separately. Thirty-eight SNPs in the *BDNF* and *BDNF-AS* loci were identified on Illumina^®^ Human OmniExpress-24 BeadChip arrays, and genotypes for the SNPs in this region were extracted from the microarray data for 597 individuals. Due to the selected quality control thresholds (minor allele frequency (MAF) > 0.01; Hardy-Weinberg Equilibrium (HWE) > 0.001), four SNPs (rs7102024, rs8192466, rs11819808, rs12291063) were excluded from the analysis. Association analysis with VR I and VR II test scores were performed for the 34 remaining SNPs using generalized linear model (GLM). To consider and eliminate Type 1 error, the significance threshold was calculated and adjusted to be able to declare the significantly associated SNPs. To calculate our *p*-value threshold, SNPs in linkage disequilibrium (LD) were considered and after estimating the effective number of independent markers (*n* = 17.4), we set the significance threshold to 0.00288 and the suggestive *p*-value as 0.05.

Results displayed in Table 2 show that none of the markers in the region of the *BDNF* and BDNF-*AS* genes were significantly associated with immediate visual memory test scores (VR I).

However, analysis of SNPs with long-term visual memory scores obtained from VR II revealed significant associations with two markers located in the *BDNF* gene (rs7103411 and rs988748) and one marker located in the *BDNF-AS* locus (rs7130131) (Table 3). Our study also identified four markers located in the *BDNF* gene and six markers located in the *BDNF-AS* gene as nominally associated with long-term visual memory, but which did not meet our corrected *p*-value threshold (0.00288). The rs7124442 and rs6265 (Val66Met) SNPs were nominally associated with the VR II and both were located in exons of the *BDNF* gene. Val66Met is a missense variant with a MAF of 0.19, and rs7124442 is a variant in the 3′untranslated region of *BDNF* with a MAF of 0.28 in our population. The nominal association with the Val66Met polymorphism in the present study population (*n* = 597) supports our earlier findings performed in a subset of this cohort (*n* = 181) [28].

**Figure 1:**
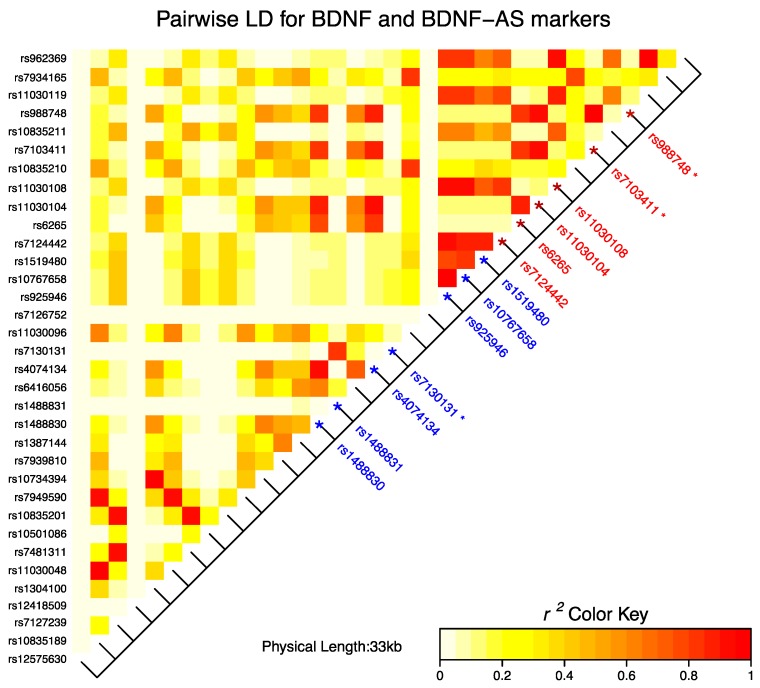
Linkage disequilibrium (LD) structure (*r*^2^ values) of *BDNF* and *BDNF-AS* markers. SNPs that are presented in the heat-map have shown significant associations with long-term visual memory. SNPs located in the *BDNF* and *BDNF-AS* genes are shown in red and blue colors, respectively. * SNPs that are significantly associated with VR II.

We calculated *r*^2^ values for the 34 SNPs in the association analysis to identify which are tightly linked in our population (Figure 1). The positions of the significant SNPs in Table 2 are shown in Figure 1. The color scale of *r*^2^ values demonstrates the degree of LD in a pairwise manner. The LD heat-map revealed that in our cohort SNPs rs925946, rs10767658, rs1519480, and rs7124442 are located in the same LD block. The *BDNF* Val66Met polymorphism is in strong LD with rs11030104, which is located in an intronic region of *BDNF*, and in moderate LD with rs1488830, rs4074134, rs7103411, and rs988748, also located in intronic regions.

**Figure 2:**
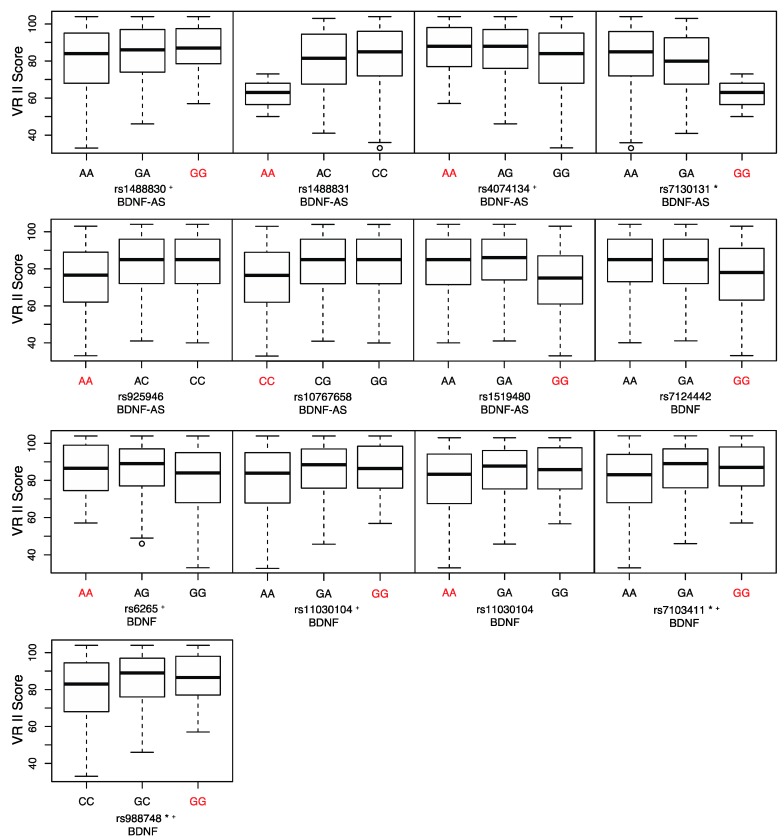
Visual Reproduction II (VR II) scores and allelic distribution of significant SNPs. Boxplots showing genotypes of the significant SNPs in *BDNF* and *BDNF-AS* genes with long-term visual memory scores. VR II was used to measure long-term visual memory. * SNPs significantly associated with VR II. ^+^ SNPs with a positive beta score. The minor alleles of the SNPs are shown in red.

We then used the VR II sub-test to measure long-term visual memory. VR II scores are normally distributed in our population and range from 33 to 104 with a mean of 82.32 (M = 84, SD = 15.52). In Figure 2, allelic distributions of the associated SNPs are presented with the minor allele shown in red. Seven of the SNPs have negative beta scores (Table 3) and six SNPs have a positive beta score (labelled with a star in Figure 2). The SNPs that have positive beta scores are in LD with the Val66Met polymorphism, which also has a positive beta score. Thus, we find that the minor A allele of rs6265 is associated with better long-term visual memory, as we previously reported in Yogeetha et al. [28].

## 3. Discussion

In this study, we performed a targeted analysis of the *BDNF* and *BDNF-AS* genes to investigate the correlation of SNPs with immediate and long-term visual memory in a healthy cohort. As presented in Table 1, our cohort was comprised of mostly females and young adults (age group 16–25), with one quarter of the participants reported as non-Caucasians. Due to the characteristics of this cohort, we included age, gender, and population structure as well as IQ to the analysis as co-variates in order to minimize their effect on the study and to focus on the association of the loci of interest and visual memory. While we found no significant associations with immediate visual memory as assessed by VR I, we identified a number of SNPs that were associated with long-term visual memory (assessed by VR II): six SNPs in *BDNF* (rs7124442, rs6265, rs11030104, rs11030108, rs7103411, and rs988748), and seven SNPs in *BDNF-AS* (rs1488830, rs1488831, rs4074134, rs7130131, rs925946, rs10767658, and rs1519480). Three of these SNPs (rs7103411, rs988748, and rs7130131) were significantly associated after correction for multiple testing, while the others showed suggestive association. Although only nominally significant in this study, the result and the effect of the rs6265 Val66Met polymorphism was shown to be consistent with our previous findings [28]. Here, we have extended our investigation to SNPs in both the *BDNF* and *BDNF-AS* genes. The relationship between the LD heat-map and the beta scores of the correlated SNPs shows that markers with positive beta scores (rs1488830, rs4074134, rs6265, rs11030104, rs7103411, and rs988748) are closely linked and are in strong LD with rs6265. The minor alleles of these SNPs are associated with better long-term visual memory performance, whereas minor alleles of the rs1488831, rs7130131, rs925946, rs10767658, rs1519480, rs7124442, and rs11030108 were found to be negatively associated.

In 2011, O’Bryant et al. investigated the association of serum BDNF levels and immediate visual memory in an Alzheimer’s disease (AD) case/control study using the VR I memory test. They reported a significant negative association of serum BDNF levels with immediate visual memory scores in the AD patients and suggested that upregulation of BDNF may be a compensatory mechanism in AD [29]. Inhibition of *BDNF-AS* upregulates *BDNF* [17,20] although the mechanism is not well understood. In addition to the differences between ours and the O’Bryant study (e.g., healthy cohort vs. AD case/control cohort and expression analysis vs. genetic association analysis), we do not know how the majority of SNPs in our study might affect BDNF levels or function in brain regions or in serum to allow comparison of results.

Beste et al. tested whether the *BDNF* Val66Met polymorphism was associated with sensory memory (immediate visual memory) in 211 individuals and reported that Met carriers showed significantly less time stability of the information stored compared to Val carriers for iconic memory (immediate visual memory) in their healthy cohort [30]. However, in our healthy cohort, we found no association with immediate visual memory. The contradictory results might be due to the differences in memory measurements (e.g., a computer-based speed tracking test vs. paper-based drawings of the shown geometrical patterns) and/or lack of power. The Beste et al. study did not investigate rs6265 with respect to long-term visual memory effects.

Our data failed to show any associations with VR I test score and the *BDNF* and *BDNF-AS* markers, whereas the VR II showed associations with the 13 of the variants, three of which were signification after multiple testing. It should be noted that although these two memory phenotypes require the participant to memorize and draw the same geometrical patterns, they evaluate two distinct memory types. The delay component in the VR II requires the functions of long-term memory since the immediate memory is not capable of holding pieces of information for longer periods of time. Our results, strengthen the concept that genetic variation plays a role in the different functions of SM, STM, and LTM. One of the biggest differences between STM and LTM is due to its capacity of holding information for longer periods of time, thus LTM requires consolidation through gene expression and protein synthesis, whereas STM does not [31,32,33]. It has been reported that activity-induced *BDNF* expression influences several forms of LTM [34], and Bekinschtein et al. have shown that *BDNF* is essential in memory persistence in rats [16,35]. *BDNF-AS* has a role in regulation of *BDNF* in humans [17,20,21], but is not present in rats.

There have been only a few studies investigating the association between *BDNF-AS* and cognition. In 2010, Cathomas et al. tested the association between markers in the *BDNF* and *BDNF-AS* loci with episodic memory using a fine-mapping approach. Their results presented five markers (rs7125904, rs10835190, rs7127239, rs6265, and rs10835218) that were nominally associated with the episodic memory phenotype [36], of which two overlap with our set of SNPs (i.e., rs6265 and rs7127239). The rs6265 SNP is nominally associated in our study with a *p*-value of 0.006, however, rs7127239 showed no association (*p*-value = 0.079). Several studies have investigated the association of *BDNF* SNPs with episodic memory which aligns with long-term visual memory; these studies focused on the Val66Met polymorphism, with the majority finding that the Met allele was associated with poorer episodic memory scores [11,37,38,39,40]. However, a number of studies have reported a link between the Met allele and better episodic memory scores [28,41,42], corresponding to our findings for long-term visual memory. These contradictory results may be a result of the complex nature of memory phenotyping (i.e., using different memory tests), which might utilize different regions and the functions of the brain resulting in differences in what is being measured. Mandelman and Grigorenko also emphasized in their meta-analysis of 23 publications, comprising 31 independent samples and 7095 individuals, that the results of the published research on the Val6Met polymorphism and cognition were inconsistent, and they failed to identify any significant associations [27]. The authors suggest various reasons for the conflict in the literature such as: small sample size of some of the studies, employment of different tasks to measure similar traits, variations due to different ethnicities, diverse health statuses of the cohorts, and neglect of the effect of a combination of alleles with the focus only on the Val66Met polymorphism [27]. Moreover, Harrisberger et al. also commented on the contradicting results on hippocampal volume with respect to *BDNF* polymorphisms, emphasizing the limited power of many studies, a lack of correction for multiple testing, variations in ethnicities, and failure to exclude environmental factors [26]. The authors of both meta-analyses indicated that further studies are required in the field for better homogeneity.

Our present analysis revealed three significant SNPs (rs7103411, rs988748, and rs7130131) and 10 variants suggestively associated (rs7124442, rs6265, rs11030104, rs11030108, rs1488830, rs1488831, rs4074134, rs925946, rs10767658, and rs1519480) with long-term visual memory. Association analysis was conducted with 597 individuals and adjusted for age, gender, ethnicity, and IQ. Sample size is highly important while investigating complex traits such as memory; therefore, SNPs that failed to pass the significance threshold are likely a result of the sample size, and the addition of these covariates can reduce power in association studies. Six of the associated SNPs (rs1488830, rs1488831, rs4074134, rs7130131, rs925946, and rs10767658) in the *BDNF-AS* gene have not previously been reported in memory related studies. Our study is the first to report a significant association between the *BDNF-AS* SNP rs7130131 and long-term visual memory. The *BDNF-AS* polymorphisms rs925946 and rs1519480, and *BDNF* rs7124442, rs11030104, rs11030108, rs7103411, and rs988748 have been previously investigated in relation to other cognitive functions. Honea et al. studied the rs925946, rs11030104, and rs11030108 SNPs and reported a significant association between rs11030108 and measures of cognitive decline in an Alzheimer’s cohort [43]. rs7124442 and rs1519480 have been reported as being significantly associated with general cognitive intelligence post-brain injury [44]. In an investigation of cognitive performances in patients with brain tumors, rs11030104 showed significant association with higher long-term verbal memory [45], and a working memory study revealed that rs7103411 is linked to poorer cognitive performance in an elderly population [46]. Finally, rs988748 was included in a study to investigate cognitive performance in a healthy Polish cohort, but was later excluded as it was out of HWE [47]. Thus, several studies suggest that *BDNF* and *BDNF-AS* SNPs may influence a range of cognitive functions, and further research is needed to dissect their roles in the various aspects of memory and learning performance.

We performed a targeted analysis of SNPs in the *BDNF* and *BDNF-AS* genes, focusing on the correlations between SNP genotypes with immediate and long-term visual memory performance. We discovered several significant associations between the SNPs in the region of interest and long-term visual memory, which includes markers not previously reported to be involved in memory. Overall, our findings establish new potential targets for future studies and enhance our knowledge on these important loci in cognition and learning.

## 4. Materials and Methods

### 4.1. Subjects

Individuals (*n* = 597) from the Brisbane and Gold Coast areas of South-East Queensland in Australia were recruited through advertisements. Participation was excluded for individuals with a history of psychiatric disorder or head injury to maintain a representative sample of cognitive and memory ability without additional complications. The study was approved by the Griffith University (MSC/01/09/HREC) and Queensland University of Technology, Human Research and Ethics (1300000486) Committees. Written informed consent was provided by all participants prior to any study activities.

### 4.2. Phenotyping

All participants were assessed individually and under the same conditions, in a quiet and well-lit room by the same examiner.

#### 4.2.1. Visual Memory

Participants undertook Visual Reproduction test I and II to gauge their memory status. Visual reproduction (VR) is a subtest of Wechsler Memory Scale—Fourth Edition (WMS IV), which is a derived version of Wechsler Adult Intelligence Scale III; however, this test evaluates memory rather than intelligence. The visual reproduction test measures visual memory using Visual Reproduction I (VR I) to assess iconic memory and STM. The Visual Reproduction II (VR II) test assesses long-term visual memory using a delay task. The test examines individuals by asking them to draw a design that they have been shown both immediately and following a 20-min delay. This memory test is used clinically for patients with mild to severe memory impairment [48].

#### 4.2.2. Intelligence Quotient (IQ)

The intelligence quotient (IQ) was measured using subsets of the Wechsler Abbreviated Scale of Intelligence (WASI) IQ test, created in 1955 by David Wechsler, which is a well-established IQ test for measuring adult intelligence [49]. The vocabulary and matrix reasoning subsets of WASI were completed in this study to estimate the IQ of participants.

### 4.3. Genotyping

Saliva samples were collected from each participant immediately after completion of the memory tests using Oragene^®^ DNA Self-Collection kits (DNA Genotek Inc., Ottawa, ON, Canada). DNA was extracted from whole saliva samples by using the kit and protocol of the same manufacturer. SNPs in loci of interest were extracted from available genotype data obtained from Illumina^®^ Human OmniExpress-24 BeadChip arrays (Illumina Inc., San Diego, CA, USA). SNPs located in the *BDNF* and *BDNF-AS* gene loci were included in the study. 

### 4.4. Statistical Analysis

All descriptive statistics were carried out using The R Program for Statistical Computing (v3.2.2) [50]. Visual memory tests and IQ were investigated for their correlation using Pearson’s *r* test. Quality control of the genotype data and association analyses were conducted using PLINK (v1.09) [51]. Population structure was studied using The R Program and KING (v1.9) [52], and a population structure inference and principal component analysis (PCA) were undertaken to consider population structure. Quality control thresholds for the analysis were determined as follows: minor allele frequency and Hardy-Weinberg equilibrium were set to higher than 0.01 and 0.001, respectively, and completed for 38 markers. Generalized linear model analysis was carried out with the statistical significance level of *p*-value of less than 0.05 for each memory phenotype individually to identify associations. Age, sex, IQ, Principle Component 1 (PC1), and Principle Component 2 (PC2) were considered as covariates in the analysis. Association analysis was performed for 34 BDNF and BDNF-AS SNPs from the Illumina OmniExpress BeadChip assay to estimate genotypic effects on memory status. To omit type I error, we have calculated the statistical significance threshold using Genetic type 1 Error Calculator (GEC) (v0.2) [53]. An α-level of 0.00288 was set as the statistical significance threshold and 0.05 as a threshold of suggestive significance.

## 5. Conclusions

In our memory cohort, we found significant associations between SNPs in the *BDNF* and *BDNF-AS* gene loci and long-term visual memory, as well as several nominally associated markers including BDNF Val66Met polymorphism. Some of our findings provide the first evidence of associations between these particular SNPs and memory in healthy individuals and support the role of *BDNF* in human memory. More extensive studies on larger cohorts are necessary to clarify the role of Val66Met polymorphisms as well as others in *BDNF* and its antisense gene *BDNF-AS* in the field of memory.

## Figures and Tables

**Table 1:** Demographics of the memory cohort.

Variable	Participants (*n* = 597) *n* (%)
Age group	
16–25	465 (77.89)
26–35	79 (13.23)
36–45	36 (6.03)
46–55	10 (1.68)
56–65	7 (1.17)
**Gender**	
Male	171 (28.64)
Female	426 (71.36)
**Ethnicity**	
Caucasian	446 (74.71)
Other	151 (25.29)

**Table 2:** Immediate visual memory score (Visual Reproduction I) association with *BDNF* and *BDNF-AS* single nucleotide polymorphisms (SNPs).

Gene	SNP	*n*	β	*t*	*p*
*BDNF-AS*	rs12575630	597	0.1069	0.04352	0.9653
*BDNF-AS*	rs10835189	595	−0.06228	−0.1376	0.8906
*BDNF-AS*	rs7127239	597	−0.1875	−0.3287	0.7425
*BDNF-AS*	rs12418509	596	−1.384	−0.6866	0.4926
*BDNF-AS*	rs1304100	597	0.214	0.4083	0.6832
*BDNF-AS*	rs11030048	597	−0.06239	−0.1388	0.8897
*BDNF-AS*	rs7481311	596	−0.2981	−0.5172	0.6052
*BDNF-AS*	rs10501086	597	−1.164	−1.199	0.2311
*BDNF-AS*	rs10835201	593	−0.1955	−0.3434	0.7314
*BDNF-AS*	rs7949590	597	0.08822	0.197	0.8439
*BDNF-AS*	rs10734394	593	0.2687	0.5155	0.6064
*BDNF-AS*	rs7939810	596	0.4341	0.9243	0.3557
*BDNF-AS*	rs1387144	597	0.2581	0.5525	0.5808
*BDNF-AS*	rs1488830	596	0.5671	1.04	0.2986
*BDNF-AS*	rs1488831	596	−1.65	−1.717	0.08646
*BDNF-AS*	rs6416056	596	0.0166	0.03337	0.9734
*BDNF-AS*	rs4074134	597	0.5361	0.9848	0.3251
*BDNF-AS*	rs7130131	597	−1.862	−1.907	0.05703
*BDNF-AS*	rs11030096	592	0.2432	0.5315	0.5953
*BDNF-AS*	rs7126752	595	−1.593	−0.8951	0.3711
*BDNF-AS*	rs925946	597	−0.6891	−1.33	0.184
*BDNF-AS*	rs10767658	590	−0.7212	−1.385	0.1666
*BDNF-AS*	rs1519480	597	−0.8752	−1.759	0.07906
*BDNF*	rs7124442	596	−0.7843	−1.536	0.125
*BDNF*	rs6265	597	0.01941	0.03417	0.9728
*BDNF*	rs11030104	596	0.5505	1.005	0.3155
*BDNF*	rs11030108	597	−0.6835	−1.363	0.1734
*BDNF*	rs10835210	578	0.1817	0.3887	0.6977
*BDNF*	rs7103411	595	0.5021	0.9257	0.355
*BDNF*	rs10835211	597	−0.1405	−0.2543	0.7994
*BDNF*	rs988748	594	0.3864	0.7136	0.4758
*BDNF*	rs11030119	597	−0.56	−1.093	0.2747
*BDNF*	rs7934165	594	0.3138	0.7005	0.4839
*BDNF*	rs962369	597	−0.469	−0.9112	0.3626

β: β score, *t*: T-statistic, *p*: *p*-value.

**Table 3:** Long-term visual memory score (VR II) association with *BDNF* and *BDNF-AS* SNPs.

Gene	SNP	*n*	β	*t*	*p*
*BDNF-AS*	rs12575630	597	−3.318	−0.7429	0.4578
*BDNF-AS*	rs10835189	595	0.02254	0.02741	0.9781
*BDNF-AS*	rs7127239	597	−1.822	−1.761	0.07878
*BDNF-AS*	rs12418509	596	−2.796	−0.7637	0.4454
*BDNF-AS*	rs1304100	597	1.733	1.823	0.06885
*BDNF-AS*	rs11030048	597	0.09195	0.1125	0.9105
*BDNF-AS*	rs7481311	596	−1.976	−1.891	0.05916
*BDNF-AS*	rs10501086	597	−1.619	−0.9167	0.3597
*BDNF-AS*	rs10835201	593	−1.75	−1.702	0.08923
*BDNF-AS*	rs7949590	597	0.213	0.2615	0.7938
*BDNF-AS*	rs10734394	593	1.668	1.762	0.07856
*BDNF-AS*	rs7939810	596	1.35	1.582	0.1142
*BDNF-AS*	rs1387144	597	0.4843	0.5702	0.5688
***BDNF-AS***	**rs1488830**	**596**	**2.267**	**2.294**	**0.02213**
***BDNF-AS***	**rs1488831**	**596**	**−4.256**	**−2.442**	**0.0149**
*BDNF-AS*	rs6416056	596	1.19	1.317	0.1884
***BDNF-AS***	**rs4074134**	**597**	**2.751**	**2.795**	**0.005352**
***BDNF-AS***	**rs7130131**	**597**	**−5.299**	**−2.998**	0.002835 *
*BDNF-AS*	rs11030096	592	0.01831	0.022	0.9825
*BDNF-AS*	rs7126752	595	−2.312	−0.7149	0.4749
***BDNF-AS***	**rs925946**	**597**	**−2.275**	**−2.423**	**0.0157**
***BDNF-AS***	**rs10767658**	**590**	**−2.211**	**−2.343**	**0.01945**
***BDNF-AS***	**rs1519480**	**597**	**−2.669**	**−2.964**	**0.003164**
***BDNF***	**rs7124442**	**596**	**−2.268**	**−2.453**	**0.01445**
***BDNF***	**rs6265**	**597**	**2.827**	**2.754**	**0.006071**
***BDNF***	**rs11030104**	**596**	**2.763**	**2.79**	**0.005448**
***BDNF***	**rs11030108**	**597**	**−1.909**	**−2.097**	**0.03641**
*BDNF*	rs10835210	578	−0.2024	−0.2395	0.8108
***BDNF***	**rs7103411**	**595**	**3.099**	**3.167**	0.001618 *
*BDNF*	rs10835211	597	−0.797	−0.7934	0.4279
***BDNF***	**rs988748**	**594**	**2.943**	**3.013**	0.002701 *
*BDNF*	rs11030119	597	−1.799	−1.936	0.05339
*BDNF*	rs7934165	594	−0.2096	−0.2574	0.7969
*BDNF*	rs962369	597	−1.753	−1.877	0.06097

*p*-Values in bold are associated with long-term visual memory (<0.05), *p*-values in bold and with a star (*) are significantly associated with long-term visual memory (0.00288) after corrections for multiple testing. β: β score, *t*: T-statistic, *p*: *p*-value.

## Abbreviations

BDNF	Brain-Derived Neurotrophic Factor
BDNF-AS	BDNF Antisense RNA
GEC	Genetic Type 1 error calculator
GLM	Generalized Linear Model
HWE	Hardy-Weinberg Equilibrium
IQ	Intelligence quotient
LD	Linkage Disequilibrium
LTM	Long-term Memory
MAF	Minor Allele Frequency
PC1	Principle Component 1
PC2	Principle Component 2
PCA	Principle Component Analysis
proBDNF	Precursor Protein of BDNF
SM	Sensory Memory
SNP	Single Nucleotide Polymorphism
STM	Short-term Memory
VR	Visual Reproduction
VR I	Visual Reproduction I
VR II	Visual Reproduction II
WASI	Wechsler Abbreviated Scale of Intelligence
WMS IV	Wechsler Memory Scale—Fourth Edition

## References

[1] Kovas Y., Plomin R. (2006). Generalist genes: Implications for the cognitive sciences. Trends Cogn. Sci..

[2] Kandel E. (2006). Biology of the mind. Newsweek.

[3] Healy A.F., McNamara D.S. (1996). Verbal learning and memory: Does the modal model still work?. Ann. Rev. Psychol..

[4] Ogmen H., Herzog M.H. (2016). A new conceptualization of human visual sensory-memory. Front. Psychol..

[5] Todd J.J., Marois R. (2004). Capacity limit of visual short-term memory in human posterior parietal cortex. Nature.

[6] Craik F.I., Lockhart R.S. (1972). Levels of processing: A framework for memory research. J. Verbal Learn. Verbal Behav..

[7] Tulving E., Donaldson W., Bower G.H., Office of Naval Research United States (1972). Organization of Memory.

[8] Cunha C., Brambilla R., Thomas K.L. (2010). A simple role for BDNF in learning and memory?. Front. Mol. Neurosci..

[9] Chen D.Y., Bambah-Mukku D., Pollonini G., Alberini C.M. (2012). Glucocorticoid receptors recruit the caMKIIα-BDNF-CREB pathways to mediate memory consolidation. Nat. Neurosci..

[10] Bekinschtein P., Cammarota M., Izquierdo I., Medina J.H. (2008). BDNF and memory formation and storage. Neuroscientist.

[11] Hariri A.R., Goldberg T.E., Mattay V.S., Kolachana B.S., Callicott J.H., Egan M.F., Weinberger D.R. (2003). Brain-derived neurotrophic factor Val^66^Met polymorphism affects human memory-related hippocampal activity and predicts memory performance. J. Neurosci..

[12] Lu B., Nagappan G., Lu Y. (2014). BDNF and synaptic plasticity, cognitive function, and dysfunction. Handb. Exp. Pharmacol..

[13] Martin S.J., Grimwood P.D., Morris R.G. (2000). Synaptic plasticity and memory: An evaluation of the hypothesis. Ann. Rev. Neurosci..

[14] Silva A.J. (2003). Molecular and cellular cognitive studies of the role of synaptic plasticity in memory. J. Neurobiol..

[15] Pastalkova E., Serrano P., Pinkhasova D., Wallace E., Fenton A.A., Sacktor T.C. (2006). Storage of spatial information by the maintenance mechanism of LTP. Science.

[16] Bekinschtein P., Cammarota M., Katche C., Slipczuk L., Rossato J.I., Goldin A., Izquierdo I., Medina J.H. (2008). BDNF is essential to promote persistence of long-term memory storage. Proc. Natl. Acad. Sci. USA.

[17] Pruunsild P., Kazantseva A., Aid T., Palm K., Timmusk T. (2007). Dissecting the human BDNF locus: Bidirectional transcription, complex splicing, and multiple promoters. Genomics.

[18] Seidah N.G., Benjannet S., Pareek S., Chretien M., Murphy R.A. (1996). Cellular processing of the neurotrophin precursors of NT3 and BDNF by the mammalian proprotein convertases. FEBS Lett..

[19] Lu B., Pang P.T., Woo N.H. (2005). The yin and yang of neurotrophin action. Nat. Rev. Neurosci..

[20] Modarresi F., Faghihi M.A., Lopez-Toledano M.A., Fatemi R.P., Magistri M., Brothers S.P., van der Brug M.P., Wahlestedt C. (2012). Inhibition of natural antisense transcripts in vivo results in gene-specific transcriptional upregulation. Nat. Biotechnol..

[21] Jin T., Zhang H., Yang Q., Li L., Ouyang Y., Yang M., Wang F., Wang Z., Zhang J., Yuan D. (2016). The relationship between polymorphisms of *BDNFOS* and *BDNF* genes and heroin addiction in the han chinese population. J. Gene Med..

[22] Egan M.F., Kojima M., Callicott J.H., Goldberg T.E., Kolachana B.S., Bertolino A., Zaitsev E., Gold B., Goldman D., Dean M. (2003). The BDNF Val^66^Met polymorphism affects activity-dependent secretion of BDNF and human memory and hippocampal function. Cell.

[23] Dincheva I., Glatt C.E., Lee F.S. (2012). Impact of the BDNF Val66Met polymorphism on cognition: Implications for behavioral genetics. Neuroscientist.

[24] Hashimoto T., Fukui K., Takeuchi H., Yokota S., Kikuchi Y., Tomita H., Taki Y., Kawashima R. (2016). Effects of the BDNF Val^66^Met polymorphism on gray matter volume in typically developing children and adolescents. Cereb. Cortex.

[25] Harrisberger F., Spalek K., Smieskova R., Schmidt A., Coynel D., Milnik A., Fastenrath M., Freytag V., Gschwind L., Walter A. (2014). The association of the BDNF Val^66^Met polymorphism and the hippocampal volumes in healthy humans: A joint meta-analysis of published and new data. Neurosci. Biobehav. Rev..

[26] Harrisberger F., Smieskova R., Schmidt A., Lenz C., Walter A., Wittfeld K., Grabe H.J., Lang U.E., Fusar-Poli P., Borgwardt S. (2015). BDNF Val66Met polymorphism and hippocampal volume in neuropsychiatric disorders: A systematic review and meta-analysis. Neurosci. Biobehav. Rev..

[27] Mandelman S.D., Grigorenko E.L. (2012). BDNF Val66Met and cognition: All, none, or some? A meta-analysis of the genetic association. Genes Brain Behav..

[28] Yogeetha B.S., Haupt L.M., McKenzie K., Sutherland H.G., Okolicsyani R.K., Lea R.A., Maher B.H., Chan R.C., Shum D.H., Griffiths L.R. (2013). BDNF and TNF-α polymorphisms in memory. Mol. Biol. Rep..

[29] O’Bryant S.E., Hobson V.L., Hall J.R., Barber R.C., Zhang S., Johnson L., Diaz-Arrastia R. (2011). Serum brain-derived neurotrophic factor levels are specifically associated with memory performance among alzheimer’s disease cases. Dement. Geriatr. Cogn. Disord..

[30] Beste C., Schneider D., Epplen J.T., Arning L. (2011). The functional BDNF Val66Met polymorphism affects functions of pre-attentive visual sensory memory processes. Neuropharmacology.

[31] McGaugh J.L. (2000). Memory—A century of consolidation. Science.

[32] Lamprecht R., LeDoux J. (2004). Structural plasticity and memory. Nat. Rev.Neurosci..

[33] Bailey C.H., Kandel E.R., Si K. (2004). The persistence of long-term memory: A molecular approach to self-sustaining changes in learning-induced synaptic growth. Neuron.

[34] Lu Y., Christian K., Lu B. (2008). BDNF: A key regulator for protein synthesis-dependent ltp and long-term memory?. Neurobiol. Learn. Mem..

[35] Bekinschtein P., Cammarota M., Igaz L.M., Bevilaqua L.R., Izquierdo I., Medina J.H. (2007). Persistence of long-term memory storage requires a late protein synthesis- and BDNF-dependent phase in the hippocampus. Neuron.

[36] Cathomas F., Vogler C., Euler-Sigmund J.C., de Quervain D.J., Papassotiropoulos A. (2010). Fine-mapping of the brain-derived neurotrophic factor (*BDNF*) gene supports an association of the Val^66^Met polymorphism with episodic memory. Int. J. Neuropsychopharmacol..

[37] Dempster E., Toulopoulou T., McDonald C., Bramon E., Walshe M., Filbey F., Wickham H., Sham P.C., Murray R.M., Collier D.A. (2005). Association between BDNF Val^66^Met genotype and episodic memory. Am. J. Med. Genet. B Neuropsychiatr. Genet..

[38] Goldberg T.E., Iudicello J., Russo C., Elvevag B., Straub R., Egan M.F., Weinberger D.R. (2008). BDNF Val^66^Met polymorphism significantly affects *d′* in verbal recognition memory at short and long delays. Biol. Psychol..

[39] Tan Y.L., Zhou D.F., Cao L.Y., Zou Y.Z., Wu G.Y., Zhang X.Y. (2005). Effect of the BDNF Val^66^Met genotype on episodic memory in schizophrenia. Schizophr. Res..

[40] Bombardier A., Beauchemin M., Gosselin N., Poirier J., De Beaumont L. (2016). Altered episodic memory in introverted young adults carrying the BDNF_Met_ allele. Int. J. Mol. Sci..

[41] Harris S.E., Fox H., Wright A.F., Hayward C., Starr J.M., Whalley L.J., Deary I.J. (2006). The brain-derived neurotrophic factor Val66Met polymorphism is associated with age-related change in reasoning skills. Mol. Psychiatry.

[42] Gajewski P.D., Hengstler J.G., Golka K., Falkenstein M., Beste C. (2011). The met-allele of the BDNF Val^66^Met polymorphism enhances task switching in elderly. Neurobiol. Aging.

[43] Honea R.A., Cruchaga C., Perea R.D., Saykin A.J., Burns J.M., Weinberger D.R., Goate A.M., Alzheimer’s Disease Neuroimaging, I. (2013). Characterizing the role of brain derived neurotrophic factor genetic variation in alzheimer’s disease neurodegeneration. PLoS ONE.

[44] Rostami E., Krueger F., Zoubak S., Dal Monte O., Raymont V., Pardini M., Hodgkinson C.A., Goldman D., Risling M., Grafman J. (2011). BDNF polymorphism predicts general intelligence after penetrating traumatic brain injury. PLoS ONE.

[45] Correa D.D., Satagopan J., Cheung K., Arora A.K., Kryza-Lacombe M., Xu Y., Karimi S., Lyo J., DeAngelis L.M., Orlow I. (2016). COMT, BDNF, and DTNBP1 polymorphisms and cognitive functions in patients with brain tumors. Neuro Oncol..

[46] Brooks S.J., Nilsson E.K., Jacobsson J.A., Stein D.J., Fredriksson R., Lind L., Schioth H.B. (2014). BDNF polymorphisms are linked to poorer working memory performance, reduced cerebellar and hippocampal volumes and differences in prefrontal cortex in a swedish elderly population. PLoS ONE.

[47] Wilkosc M., Szalkowska A., Skibinska M., Zajac-Lamparska L., Maciukiewicz M., Araszkiewicz A. (2016). *BDNF* gene polymorphisms and haplotypes in relation to cognitive performance in polish healthy subjects. Acta Neurobiol. Exp..

[48] Brooks B.L., Holdnack J.A., Iverson G.L. (2011). Advanced clinical interpretation of the WAIS-IV and WMS-IV: Prevalence of low scores varies by level of intelligence and years of education. Assessment.

[49] Kaufman A.S., Lichtenberger E.O., McLean J.E. (2001). Two- and three-factor solutions of the WAIS-III. Assessment.

[50] R Core Team (2017). R: A Language and Environment for Statistical Computing. https://www.r-project.org/.

[51] Purcell S., Neale B., Todd-Brown K., Thomas L., Ferreira M.A.R., Bender D., Maller J., Sklar P., de Bakker P.I.W., Daly M.J. (2007). PLINK: A tool set for whole-genome association and population-based linkage analyses. Am. J. Hum. Genet..

[52] Manichaikul A., Mychaleckyj J.C., Rich S.S., Daly K., Sale M., Chen W.M. (2010). Robust relationship inference in genome-wide association studies. Bioinformatics.

[53] Li M.X., Yeung J.M.Y., Cherny S.S., Sham P.C. (2012). Evaluating the effective numbers of independent tests and significant *p*-value thresholds in commercial genotyping arrays and public imputation reference datasets. Hum. Genet..

